# The Role of Digital Tools in Meeting the Needs of Adults With Tourette Syndrome: A Human-Centered Design Approach

**DOI:** 10.2196/78328

**Published:** 2026-02-19

**Authors:** Sarah M Harmon, Hannah E Reese

**Affiliations:** 1 Department of Computer Science, Bowdoin College, 255 Maine St, Brunswick, ME, 04011, United States, 1 207-798-4286; 2 Department of Psychology, Bowdoin College, Brunswick, ME, United States

**Keywords:** Tourette syndrome, tics, tic disorders, user experience, user research, accessibility, qualitative analysis, online, internet, apps, mobile phone

## Abstract

**Background:**

Individuals with tic disorders (TDs) have access to a small but growing number of digital tools (such as apps and websites) for tic management and support. While prior work has shown promise for these tools, they have traditionally been designed by researchers first and evaluated by members of the TD community after tool development is complete. A human-centered design process targeting this domain has the potential to reveal new insights relevant to the development of future tools. We seek to establish a preliminary understanding of how the TD community uses and perceives current resources for tic management and support as well as their overall concerns and needs in this area.

**Objective:**

This study aimed to explore the design potential of future digital tools for helping adults manage their tics by gathering an initial set of needs and requirements from adult members of the TD community in the United States.

**Methods:**

An online survey was distributed via TD community groups and also via TD clinicians and researchers in the United States. The survey contained a combination of dichotomous, multiple-choice, and open-ended questions, with opportunities for participants to specify how they currently receive support, rank their preferred features and requirements, and express their needs and concerns relevant to future work. Qualitative responses were analyzed with inductive thematic analysis.

**Results:**

Most respondents typically sought answers from digital platforms first (124/158, 78.5%) when confronting a question about their tics. Even so, only 18.4% (29/158) reported having previously used a digital tool to help with their tics or any other aspect of their health. Simultaneously, 88.9% (136/153) indicated that they would be very (81/153, 52.9%) or somewhat (55/153, 35.9%) likely to use a digital tool designed for adults with tics. Of those listing concerns (42/158, 26.6%), common reported concerns included the tool being too time-consuming, difficult to use, or generally not meeting accessibility standards. When asked to rank the one feature of a digital tool that they believed to be most important, tic monitoring (66/154, 42.9%) and trigger monitoring (54/154, 35.1%) were among the most popular requested features as opposed to other options, such as information gathering, reminders to practice a therapeutic skill or take medicine, social support, or opportunities to share their story. While screen navigation was most preferred, results indicated that a multimodal design overall would support the most users.

**Conclusions:**

Our study participants reported a lack of useful technology for tic management and indicated a need for accessible tools to assist in tic and trigger monitoring in particular. Other concerns included that new tools would be difficult to use or learn due to tics. Findings suggest a cautious excitement for future digital tools in this area.

## Introduction

Tourette syndrome (TS) and persistent tic disorder (collectively referred to as tic disorders or TDs) are neurodevelopmental disorders characterized by sudden, rapid, repetitive motor movements or vocalizations (ie, tics) that develop in childhood, persist for more than a year (although waxing and waning are common), and are not attributable to a substance or other medical condition [1]. Common tics include eye blinking, head jerking, sniffing, throat clearing, and simple vocalizations [2,3]. Most individuals with tics also report uncomfortable physical sensations (eg, an itch, pressure, or ache) that precede some or all of their tics and are temporarily relieved by the tics [4,5]. These premonitory sensations are thought to play a critical role in the development and maintenance of TDs [6] and are sometimes described as more distressing than the tics themselves [7]. TDs affect between 0.3% and 0.9% of the general population [8,9]. Recent reviews suggest that contrary to popular belief, tics persist into adulthood for the majority of individuals, with complete remission occurring in only 10%‐20% of individuals [10].

Although clinical guidelines for the treatment of TD and TS have been developed [11], a study by Cuenca et al [12] indicated that individuals with TDs and their allies within the TD community (including their parents) perceived health professionals as having limited knowledge about them. The authors also found that access to behavioral treatment, the recommended first-line intervention, was limited despite the fact that 76% of parents wanted the treatment to be available for their child. This is consistent with other reports documenting difficulty accessing specialty care [13], negative experiences when receiving care [14], and a global paucity of specialized TD clinics [15]. Another challenge, particularly for adults with TDs, is that because tics emerge in childhood, most of the treatments and resources are designed for younger audiences. This is unfortunate given that tics do commonly persist into adulthood [10] and adults with TDs continue to experience impairment and lower quality of life [16].

Many researchers have hoped that technology could help close this treatment gap and ensure that individuals within the TD community receive more support. As a result, a variety of ways in which technology can be used to increase access have been proposed. These approaches range from therapist-delivered online interventions (eg, telehealth services [17,18]) to stand-alone websites, apps, and wearables with no human interaction at all [19-21].

It is crucial that any such technology be designed with these users and their allied stakeholders in mind. Still, little work has been done to gather feedback from these communities prior to building tools and services. One exception might be [22], which examined user experience relevant to online support communities for TDs. There may be additional tools or support systems that could meaningfully benefit individuals with tics, but we currently lack a clear understanding of how users perceive existing technologies in this area (eg, TicVision [23]) and what they identify as their most pressing needs.

The aim of this study was to provide initial data to support a human-centered design approach for the next generation in technology for individuals with tics. To this end, we evaluated how adults with tics in the United States use and perceive available resources for tic management and support. We also included questions to better understand their reported concerns about using technology in this way, as well as what they would like this kind of technology to do for them.

## Methods

### Survey

We designed an exploratory online survey created and administered via Qualtrics to obtain rapid input from a geographically dispersed sample. The survey consisted of 17 questions pertaining to demographic information (age, gender, race, ethnicity, and state of residence), clinical characteristics (diagnostic status, age of onset, and treatment history), sources of tic-related information and support, and experiences with, preferences for, and concerns about a digital tool for managing one’s tics. We define *support* for adults with tics as “anything that helps adults with tics manage or feel better about their tics,” which could include but is not limited to treatment, accommodations at school or work, encouragement, or understanding from others. A combination of dichotomous, multiple-choice, and open-ended questions were used, including 3 conditionally displayed items to allow participants to provide more detail to a positively answered dichotomous question.

To develop the questionnaire items, we first identified domains from our research aims and specified the constructs to be measured. All authors jointly drafted and reviewed items in working sessions, resolving discrepancies by consensus. We compared our draft wording with items used in prior studies and relevant guidance, adapting where appropriate to maintain conceptual alignment and plain language. While we did not conduct a separate pilot, we performed internal usability checks (skip logic, option ordering, and readability) on desktop computers, laptop computers, tablets, and smartphones (iOS) before launch. To reduce participant burden and accommodate potential completion difficulties, most survey items were left optional. Because our approach was reach rather than depth, findings should be interpreted with this limitation in mind. We expect pilot testing and further validation of the instrument in future work.

### Recruitment

The survey was distributed to adults with motor or vocal tics who were living in the United States via an advertisement on the Tourette Association of America (TAA) website (tourette.org) and emails to state and local TAA chapters and support groups, as well as TD clinicians and researchers.

### Data and Participants

Data were collected between December of 2022 and April of 2023. The survey received 170 entries, with 2 noted duplicates. Of the 168 unique individuals who accessed the survey, 152 completed it fully (completion rate=90.5%). Responses were excluded from analysis if they indicated they did not wish to participate (n=1), a duplicate response was recorded (n=2), or they completed only the consent and eligibility section and nothing else (n=9). Data from partial completers (n=6) are included where available.

Among completers, the mean completion time was 26 minutes (SD 44 minutes; median 21 minutes, range 4‐532 minutes). The IQR was 13 minutes (IQR/median=0.65, indicating high variability). Excluded responses (n=12) and partial responses (n=6) were not included in the completion time analysis.

### Data Analysis

#### Quantitative Analysis

For categorical variables, we calculated frequencies and proportions (counts divided by the total number of responses) reported as counts and percentages. Analyses were performed in Google Sheets (Alphabet Inc).

#### Qualitative Analysis of Open-Ended Responses

We conducted an inductive thematic analysis of open-ended survey responses following Braun and Clarke’s 6-phase approach [24]. Coding and theme development were driven by the data rather than preexisting frameworks. In phase 1 (familiarization), all responses to the open-ended survey questions were read to establish initial impressions. In phase 2 (generating initial codes), each response was coded by assigning concise labels that captured the meaning of each segment of text (eg, “confidentiality,” “price,” and “time-consuming”). Coding was conducted in Google Sheets (Alphabet Inc) to organize data systematically. In phase 3 (searching for themes), related codes were grouped into broader candidate themes. For example, codes such as “boring” and “doesn’t attract attention” were combined into the candidate theme “Lack of engagement.”

In phase 4 (reviewing themes), candidate themes were reviewed against the coded data and the entire dataset to ensure coherence within themes and clear distinctions between themes. Discrepancies were resolved through discussion. In phase 5 (defining themes), we refined the scope and focus of each theme and developed clear definitions and names that captured their essence. In phase 6 (writing the report), we selected representative participant quotes for each theme to illustrate key points when reporting results. Quotes are presented with participant IDs (labeled as P1-P168), which were assigned based on what time the survey was submitted. After coding all responses, we calculated the number and proportion of participants whose comments were assigned to each theme. This allowed us to report both qualitative descriptions and quantitative distributions of concerns.

### Ethical Considerations

The study was reviewed and deemed exempt by the institutional review board at the authors’ institution (Bowdoin College) per Exemption 45 CFR 46.104(d)(2) for Educational Tests, Surveys, Interviews, or Observation of Public Behavior. A limited review was conducted to ensure that there were adequate provisions to protect the privacy of participants and to maintain the confidentiality of data. All participants completed an informed consent checkbox, and data were stored on a secure, password-protected server with no identifying information. Participants received a US $10 Amazon gift card for their participation.

## Results

### Demographic Information

Among completers and partial completers (N=158), participants were predominantly male (n=105, 66.5% male; n=49, 31.0% female; and n=4, 2.5% nonbinary) and ranged in age from 18 to 87 years (mean 29.3, SD 12.8 years). Altogether, 65.2% (n=103) of participants identified as White, 23.4% (n=37) as Black or African American, 5.1% (n=8) as more than 1 race, 3.8% (n=6) as Asian, 1.9% (n=3) as another race, and 0.6% (n=1) did not report their racial identity. In all, 6.3% (n=10) were of Hispanic origin.

Participants were asked where they currently lived as a nonrequired question. One participant came from the District of Columbia, and the remaining participants lived in 42 different states across the United States. Of those who responded (N=157), the largest overrepresentation was California (n=13, 8.3%), New Jersey (n=12, 7.6%), and Washington (n=12, 7.6%). The largest underrepresentation (n=0, 0%) was Alaska, Maine, Mississippi, Montana, North Dakota, Rhode Island, South Dakota, and Wisconsin. Given the limited literature on comparable demographic distributions, comprehensive benchmarking is not feasible. Nevertheless, our sample appears consistent with prior reports regarding high male preponderance [25,26].

### Clinical Characteristics

Among respondents (N=158), the vast majority (n=143, 90.5%) of the participants reported having been formally diagnosed with TS by a health care provider. An additional 3.8% (n=6) reported having been diagnosed with persistent or chronic motor or vocal tic disorder, while 1.9% (n=3) were diagnosed with provisional or transient tic disorder and 1.3% (n=2) with another TD (eg, tic disorder not otherwise specified). The remaining 2.5% (n=4) did not disclose that they were formally diagnosed, leaving their answer blank. We chose to include these respondents as part of our analysis because formal diagnosis is often limited by access and affordability of care [27].

Participants were asked, “What age did your tics begin?” and provided their answers in a text field. Two respondents gave a generic answer (“teens” and “toddler”), and 1 partial completer did not answer the question. Among respondents who gave an age in years (N=155), the reported median age of onset was 7 (IQR 6‐8) years. The range was between 1 and 55 years (mean 7.8, SD 4.6 years).

Participants were asked, “Have you ever received any of the following treatments for your tics?” and were asked to select any options that best fit their experience. Six partial completers did not respond. Among respondents (N=152), the majority reported receiving at least 1 type of treatment for their tics, with comprehensive behavioral intervention or habit reversal therapy being the most common (n=131, 86.2%), followed by exposure and response prevention (n=96, 63.2%), medication (n=92, 60.5%), other therapy (n=12, 7.9%), other interventions (n=4, 2.6%, self-described as meditation, anxiety management, and radio frequency), and deep brain stimulation (n=2, 1.3%).

### Sources of Information and Support

#### Information

**Figure 1.**
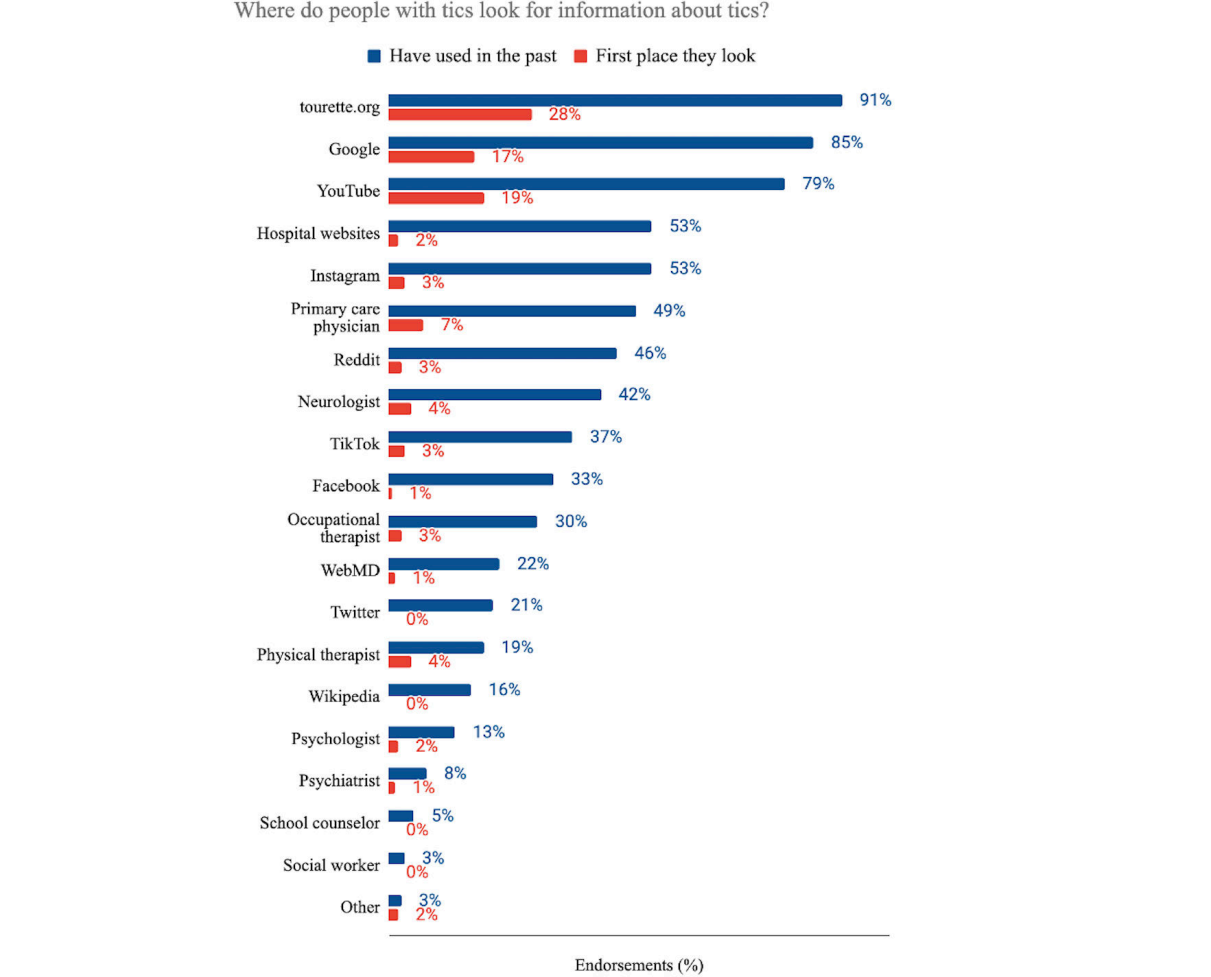
Percentage of endorsements for information sources on tics are reported as percentages (N=158) for each category: tourette.org (have used: n=143, 91%; first place they look: n=45, 28%), Google (have used: n=134, 85%; first place they look: n=27, 17%), YouTube (have used: n=125, 79%; first place they look: n=30, 19%), hospital websites (have used: n=83, 53%; first place they look: n=3, 2%), Instagram (have used: n=83, 53%; first place they look: n=5, 3%), primary care physician (have used: n=78, 49%; first place they look: n=11, 7%), Reddit (have used: n=72, 46%; first place they look: n=4, 3%), neurologist (have used: n=67, 42%; first place they look: n=7, 4%), TikTok (have used: n=58, 37%; first place they look: n=5, 3%), Facebook (have used: n=52, 33%; first place they look: n=1, 1%), occupational therapist (have used: n=47, 30%; first place they look: n=4, 3%), WebMD (have used: n=35, 22%; first place they look: n=2, 1%), Twitter (have used: n=33, 21%; first place they look: n=0, 0%), physical therapist (have used: n=30, 19%; first place they look: n=7, 4%), Wikipedia (have used: n=26, 16%; first place they look: n=0, 0%), psychologist (have used: n=21, 13%; first place they look: n=3, 2%), psychiatrist (have used: n=12, 8%; first place they look: n=2, 1%), school counselor (have used: n=8, 5%; first place they look: n=0, 0%), social worker (have used: n=5, 3%; first place they look: n=0, 0%), other (have used: n=4, 3%; first place they look: n=3, 2%). For the question posed to participants, refer to Multimedia Appendix 1 (Survey Instrument, Q1).

Participants were asked to report where they had previously sought information about tics. They were provided with 21 possible sources and also permitted to enter additional sources. Online sources were the most commonly endorsed and were the only sources that greater than 50% of the respondents selected. These included tourette.org (n=143, 90.5%), Google (n=134, 84.8%) and YouTube (n=125, 79.1%), hospital websites (eg, Mayo Clinic, n=83, 52.5%), and Instagram (n=83, 52.5%). When asked where they would first look for the answer to a current question, we saw similar patterns: the majority of respondents typically sought answers from digital platforms first (124/158, 78.5%), with tourette.org (45/158, 28.5%), YouTube (30/158, 19.0%), and Google (27/158, 17.1%) as the most commonly selected. Social media generally (45/158, 28.5%) was almost as popular as more credible sources, such as PubMed, WebMD, MEDLINE, hospital websites, or the TAA’s website (53/158, 33.5%). See Figure 1 for a complete accounting of responses.

#### Support

**Figure 2.**
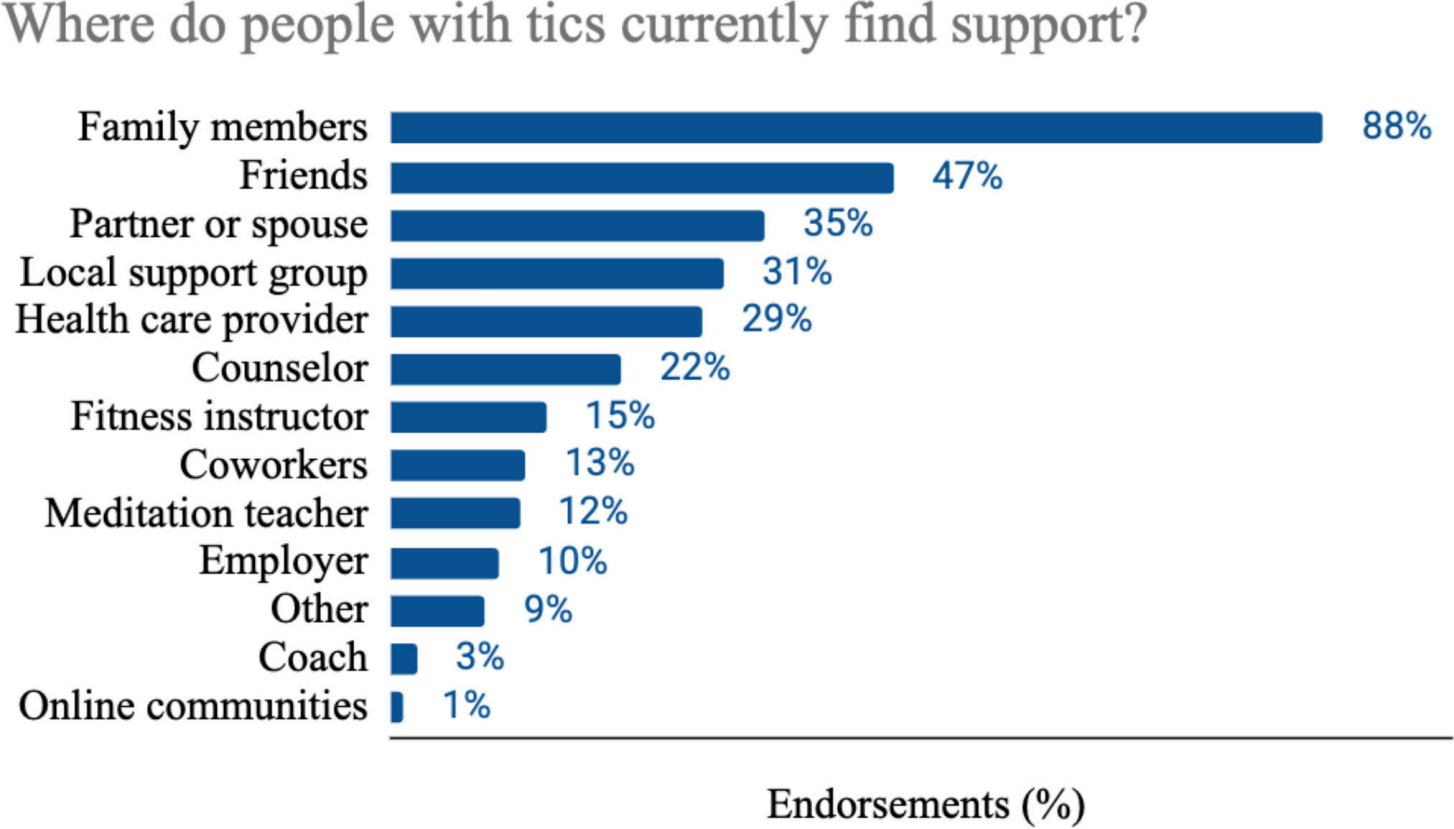
Current sources of support for individuals with tics are reported as percentages (N=156) for each category: family members (n=137, 88%), friends (n=74, 47%), partner or spouse (n=55, 35%), local support group (n=49, 31%), health care provider (n=46, 29%), counselor (n=34, 22%), fitness instructor (n=23, 15%), coworkers (n=20, 13%), meditation teacher (n=19, 12%), employer (n=16, 10%), other (n=14, 9%), coach (n=4, 3%), online communities (n=2, 1%). For the full question posed to participants, refer to Multimedia Appendix 1 (Survey Instrument, Q4. Existing Digital Tools: User Experience and Preferences).

Participants were asked a nonrequired question: “Where do you receive support for your tics? Support might include treatment, accommodations at school or work, encouragement, understanding, and more. It’s basically anything that helps you manage or feel better about your tics.” They were provided with 13 possible sources and also permitted to enter additional sources. Among respondents (N=156), intimate relationships were the primary sources of support, including family members (n=137, 87.8%), friends (n=74, 47.4%), and spouse or partner (n=55, 35.3%). Of note for this particular survey, online communities and message boards were the least endorsed sources of support (n=2, 1.3%). Figure 2 displays all responses received for this question.

Participants were also asked whether there are any forms of support that they wish they had, but do not. This question was not required to be filled in. Among completers and partial completers (N=158), 82.3% (n=130) of the participants left their answer blank and 1.9% (n=3) stated “No” or “N/A.” In total, 5.1% (n=8) of the participants named other forms of support they already had in their lives, including pets (n=4, 2.5%), gardening (n=2, 1.3%), neighbors (n=1, 0.63%), and participating in a prayer group (n=1, 0.63%). The remaining participants (n=17, 10.8%) reported a range of responses. The most commonly reported themes were better access to treatment (n=6, 3.8%) and social support specifically for adults with tics (n=5, 3.2%). On better treatment access, participants talked about access to a professional therapist, deep brain stimulation, medication without significant side effects, a version of the TicHelper online intervention designed for adults, and better treatment options generally. On social support for adults with tics, participants reported that it was difficult to get to know others with TDs as an adult, especially women. Three other themes that emerged also had to do with support group access. The remaining themes had to do with more research on treatment and support for communicating to others about their tics. A summary table of responses is shown in Table 1.

**Table 1.** Example participant responses for each theme identified in response to the question “Are there any forms of support that you wish you had, but don’t?”

Theme	Definition	Example quote
Better access to treatment	Participants wished better treatment options existed.	“I wish there was more/better options to treat them [I’m] 28 now and experiencing my tics worse than I ever had” [Participant 40].
Better support as a child	Participants wished they had access to better support as a child.	“Not much was known about this disorder when I first showed symptoms…Once there was a name to put with this, things were different” [Participant 23].
More research on treatment	Participants wished more studies were being conducted to support individuals with TDs^a^.	“I would like to see more studies on treatment options other than pharmacological” [Participant 152].
Professional support group	Participants wished for a support group led by professionals with relatable content.	“Sometimes I want an adult support group made up of professionals. But not an adult group about parenting” [Participant 14].
Social support for adults with tics	Participants wished for at least 1 other person who can relate to them as an adult with tics.	“I’d love an in-person support group for ADULTS with Tourettes where we can hang out and talk and share stories” [Participant 61].
Support for communicating to others about their tics	Participants wished for support that would help them feel more comfortable sharing with others that they had tics.	“I wish there was like, some kind of something that would’ve supported me to tell my parents about my tic development without as much stress that they would go crazy over it” [Participant 25].
Support group relevant to academics	Participants wished for a support group that could provide them with strategies and resources relevant to attending school.	“Support centered around college-related activities, including socialization, academics, and how to deal with TS’s impact on those areas of life” [Participant 72].

a TDs: tic disorders.

### Existing Digital Tools: User Experience and Preferences

#### Prior Experiences

Among respondents (N=158), 18.4% (n=29) reported having previously used a digital tool to help with their tics or any other aspect of their health. Only 1 app was described as being consistently used that was specifically designed for the TD community (BT-Coach English [28]), although 1 individual did mention telehealth and another mentioned that they had tried to make their own app for the TD community due to the lack of apps in this domain. The majority (22/29, 75.9%) of reported tools were used for mental health support and wellness. Others (4/29, 13.8%) reported using built-in productivity tools (Notes app for tic tracking, Calendar app for tic tracking or appointment management, alarms for medication reminders, speech-to-text for motor tic support, or online search for information) and fitness tracking (Fitbit or Weight Watchers). The most popular tool reported overall was the Calm app for mental health (8/29, 27.6%), followed by Sanvello (4/29, 13.8%). A number of users reported that while mental health tools were “not specifically developed to treat tics,” they did indirectly help them in other ways. A summary table of example responses is shown in Table 2.

**Table 2.** Example participant responses when asked to describe the digital tools they have used and identified themes.

Theme	Definition	Example quote
Apps specifically designed for the TD^a^ community	Participants used apps that are specifically for individuals with tics (eg, tic monitoring).	“I have used an app called The BT-Coach English, I like it because it tracks the number of tics and the strength of the need to tic” [Participant 27].
Built-in productivity tools	Participants used built-in productivity tools that are not necessarily designed for individuals with tics.	“I use alarms to remind me to take medications. My pharmacy auto fills my prescriptions. I use my cell phone calendar to manage doctor’s appointments” [Participant 3].
General health	Participants used apps designed for general health.	“Fitbit App—good, just didn’t always keep my Fitbit charged and I didn’t like that it didn’t track other kinds of exercise (other than walking) as accurately” [Participant 14].
Mental health support and wellness	Participants used apps designed for mental health and wellness.	“I have used some apps to control my negative thoughts, stress and anxiety, because there are no apps for people with tics or tourette syndrome” [Participant 54].
Unmemorable due to negative experience	Participants could not remember what app(s) they used because they had a negative experience.	“I don’t remember the name of the app, I didn’t like it, I spent a lot of time answering questions and I didn’t get anywhere. It didn’t help to improve my tics either, so I uninstalled it” [Participant 86].

a TD: tic disorder.

Most individuals reported positive results with the digital tools they had tried, with only a few (4/29, 13.8%) explicitly stating that they had a negative experience. However, many (13/29, 44.8%) mentioned that the tools they used (whether for mental health support or otherwise) were unrelated to tics. Overall, this was the most common criticism of the digital tools these individuals had tried. Other critiques included the app experiences being tedious, boring, difficult, and generally not what they expected. One user stated, “I believe I tried tracking tics long ago but it was just too frequent to keep up with it and there were no consistent triggers to record” [Participant 14].

#### Navigation

Participants were asked, “Do your tics make it difficult or frustrating for you to use a computer, smartphone, tablet, or other digital device?” Among respondents (N=158), 48.1% (n=76) responded “yes,” 48.7% (n=77) responded “no,” and 3.2% (n=5) left the question blank. Those who answered “yes” were asked a follow-up as to the nature of the difficulty or frustration. Most often, these individuals mentioned frustration with tics because they physically interrupted what they were doing such as typing or looking at a screen (26/76, 34.2%). Some further noted that using digital devices might be a trigger due to prolonged usage (5/76, 6.6%) or other, mostly unspecified reasons (7/76, 9.2%). Other difficulties included the unpredictability of tics, damage to devices (from dropping or throwing), slowness (due to having to restart or wait), needing assistance to use the device, losing concentration on tasks, submitting the wrong input (such as typos, accidentally changing the screen orientation, or moving the mouse to the wrong place), and experiencing embarrassment or discomfort. A summary table of example responses is shown in Table 3.

**Table 3.** Example participant responses when asked how their tics make it difficult or frustrating to use digital devices.

Theme	Definition	Example quote
Can’t concentrate	Participants report frustration with tics causing them to lose focus.	“Yes, tics hinder some activities such as using devices because they require...concentration.” [Participant 168]
Can’t see screen	Participants report frustration with not being able to see or read from the device.	“It makes it difficult for me to read and see the screen, because I blink and twist my neck, although it is occasionally.” [Participant 42]
Can’t sit still	Participants report frustration with being unable to sit still to use the device.	“Needing to sit still.” [Participant 153]
Device acts as a trigger	Participants report frustration that tics are exacerbated when using their digital devices.	“The brightness or light from the screens bothers my eyesight and I start to blink so I can’t concentrate on what I’m doing, so I have to stop, go somewhere else, calm down, and wait to sit down in front of the device again.” [Participant 27]
Loss of control	Participants report frustration with losing control of their body.	“I still can’t get used to the tics, it frustrates me not being able to control my body.” [Participant 163]
Manageable but still frustrating	Participants explain that their frustration has lessened over time due to tics decreasing in frequency, intensity, or both.	“My tics have decreased in intensity, from time to time they appear, but they don’t affect me as before.” [Participant 123]
Prolonged device usage	Participants report frustration that using devices for a long time will trigger tics.	“It makes me uncomfortable and stressful to use touch screens for a long time, my hands shake as part of a tic.” [Participant 33]
Prevents action	Participants report frustration with not being able to take a desired action.	“I can’t use digital tools until my tics subside.” [Participant 86]
Require assistance	Participants report frustration with tics causing them to depend on someone else to use their digital device.	“My tics make it difficult for me to use computers so much so that sometimes someone uses the computer for me and explains what they are doing.” [Participant 97]
Slip due to tics	Participants report frustration that tics cause an unintentional action.	“Sometimes when I use the mouse or the screen and I want to choose an option, my arm loses control for seconds and I select another option.” [Participant 74]
Social comparison	Participants report frustration when comparing themselves with others.	“Even when I’m calm my tics kick in and my arms shake uncontrollably, it frustrates me because I can’t use devices calmly like other people.” [Participant 70]
Social judgment	Participants report frustration that their tics cause others to treat them differently.	“The looks of the people around me make a simple daily activity like calling by phone in something very difficult.” [Participant 136]
Time-consuming	Participants report frustration with how much time it takes them to use their digital devices.	“Using a computer takes me time, and it frustrates me.” [Participant 168]
Unexpected tics	Participants report frustration with tics that appear unexpectedly with an unknown cause.	“My tics make appearances at the least expected moments, even when I’m relaxed they trigger.” [Participant 41]

Next, participants were asked, “How do you prefer to navigate on your digital devices?” Participants were presented with 5 means of navigation and instructed to rank order the choices. Means given higher rankings were assigned fewer points (eg, the first priority was given 1 point whereas the last priority was given 5 points). The overall preference of a means of navigating was computed as the mean of all ranking scores with lower scores indicating greater preference. The screen was most preferred (mean 1.97, SD 1.02), followed by mouse (mean 2.57, SD 1.26), voice commands (mean 3.01, SD 1.42), trackpad (mean 3.62, SD 1.33), and keyboard (mean 3.83, SD 1.12).

### Future Digital Tools: Perceptions and Expectations

#### Desired Functionality

Participants were asked, “A digital tool can do lots of different things. Please consider the list below and rank order the various features from most to least important.” A total of 14 possible desired functionalities for a digital tool were presented to participants. Functions given higher rankings were assigned fewer points (eg, the first priority was given 1 point whereas the last priority was given 14 points). The overall importance of a function was computed as the mean of all ranking scores with lower scores indicating greater importance. Table 4 shows the functions from most to least important. Overall, tic monitoring (66/154, 42.9%) and trigger monitoring (54/154, 35.1%) were ranked as most important to respondents.

**Table 4.** Possible functionalities of a digital tool for the tic disorder community, ranked from most (lower score) to least (higher score) important by participants in the study (N=154).

Functionality	Mean (SD)	
Tic monitoring (eg, a way to monitor how often your tics occur and see how they change over time)	2.32 (2.17)	
Trigger monitoring (eg, a way to track factors that make your tics better or worse)	2.52 (2.26)	
Mood/anxiety/stress monitoring (eg, a way to monitor your emotional states and see how they change over time)	5.35 (2.91)	
Information about tics (eg, how common they are and what causes them)	6.56 (3.09)	
Stress reduction/relaxation strategies (eg, progressive muscle relaxation and physical exercise)	7.15 (3.18)	
Tools for managing negative thoughts you may have about your tics	7.95 (3.09)	
Anger management strategies	8.02 (3.38)	
Ways to connect with others who have tics (eg, a message board)	8.12 (3.72)	
Encouragement	8.22 (3.13)	
Tips for talking about tics with others	9.33 (3.68)	
Mindfulness-based tools (eg, meditation, breathing exercises, and yoga)	9.47 (3.48)	

We examined associations between responses to Q11 (“Have you ever used a digital tool to help with your tics or any other aspect of your health?”) and how participants ranked each of the 14 features (Q6) using Pearson correlations. For Q11, we coded “Yes” responses to 1 and “No” responses to 0. For Q6, we reversed the ranking values so that a positive *r* value would denote the “Yes” group preferring a feature more than the “No” group (and a negative *r* value would denote the “Yes” group preferring a feature less than the “No” group).

Across all features, correlations were small (|r|<0.17). The strongest positive correlation was observed for the “managing negative thoughts” feature, but this effect was small and not statistically significant (*r*=0.116, *P*=.152; N=153). The strongest negative correlation was observed for the “encouragement” feature: respondents who answered “Yes” to Q11 (indicating that they had used a digital tool to help with their tics or health) tended to give “encouragement” lower priority than those who answered “No” (*r*=−0.169, *P*=.037; n=153). However, this association did not survive a Bonferroni correction for 14 tests (adjusted α=.0036). These results overall should be interpreted as exploratory.

Following this question (Q6), participants were asked, “Are there any other things that you would like a digital tool to do for you?” A number of responses elaborated on the functionalities shown in Table 4, including how to reduce tics, using tic management techniques such as Exposure Response Prevention, helping individuals “take their mind off tics” to lessen their severity, managing negative thoughts (in particular, intrusive thoughts from comorbid conditions such as obsessive-compulsive disorder), linking anxiety states with tic severity, and supporting sleep. One individual noted that tic monitoring would make their tics worse, emphasizing the need for mental health support instead. Beyond these, responses included (1) resources for the general public to help destigmatize TDs and promote acceptance, as well as tips for what others should or should not say to someone who tics; (2) reminders for individuals with TDs that it is okay to tic in public; (3) support for physical pain associated with tics; and (4) features relevant to legal rights, advocacy, and workplace accommodations, as well as the experience of discrimination in public places when tics could be disruptive.

#### Promise and Appeal

Participants were asked, “Do you think a digital tool designed for adults with tics, specifically, could be helpful?” Among respondents (N=153), the majority of participants (n=115, 75.2%) responded “Yes” (n=57, 37.3%) or “Possibly” (n=58, 37.9%). The remaining participants responded “Unsure” (n=37, 24.2%), or “Unlikely” (n=1, 0.65%). No participants responded “No.” There was a significant small positive relationship between agreement on this question (Q13) and Q11, which asked, “Have you ever used a digital tool to help with your tics or any other aspect of your health?” (Spearman ρ=0.291, *P*<.001; n=153). Results were similar using Pearson *r* (*r*=0.286, *P*<.001; N=153).

Participants were also asked, “How likely would you be to use a digital tool for adults with tics?” Among respondents (N=153), the majority of participants (n=136, 88.9%) responded “Very likely” (n=81, 52.9%) or “Somewhat likely” (n=55, 35.9%). In total, 7.8% (12/153) of the participants responded “Neither likely nor unlikely,” 3.3% (5/153) responded “Somewhat unlikely,” and no participants responded “Very unlikely.”

Finally, participants were asked, “Which type of tool would be most appealing to you?” Among respondents (N=153), the majority of participants indicated that a website and an app would both be appealing to them (n=73, 47.7%), followed by an app (n=50, 32.7%), a website (n=28, 18.3%), and neither (n=2, 1.3%). A chi-square test of independence revealed that there was no significant association between type of tool chosen as most appealing (app, website, both, or neither) and whether participants indicated that their tics made it difficult or frustrating to use a digital device (Q9), *χ*²_3_=2.99 (n=153); *P*=.394.

#### Concerns

Participants were asked, “Do you have any concerns about using a digital tool for managing your tics?” Among respondents (N=158), 70.3% (n=111) answered “No,” 26.6% (n=42) answered “Yes,” and 3.2% (n=5) left the answer blank. Of those who answered “Yes” (N=42), many were concerned about whether the digital tool would be time-consuming (n=13, 31.0%), too complicated or difficult to use (n=11, 26.2%), not accessible given their tics, such as not including the possibility for voice commands (n=9, 21.4%), and expensive (n=9, 21.4%). Other concerns included that the tool would be ineffective or not meet their expectations (6/42, 14.3%), not allow another person to help them use it (3/42, 7.1%), be boring (3/42, 7.1%), trigger tics (2/42, 4.8%), not protect confidentiality (2/42, 4.8%), be difficult to learn how to use it (2/42, 4.8%), contain inaccurate information or otherwise fail to educate (2/42, 4.8%), or that they would forget to use it (1/42, 2.4%). In total, 4.8% (2/42) did not provide more detail about their concerns. A summary table of example responses is shown in Table 5.

**Table 5.** Example participant responses when asked to explain their concerns (if they had concerns) about using a digital tool for managing their tics.

Theme	Definition	Example quote
Abandonment	Participants expressed concerns that they would forget to use the digital tool.	“Remembering to use the app” [Participant 147].
Inaccessibility	Participants expressed concerns that a digital tool would be inaccessible to someone with tics.	“For me it is important that it include voice command, so I don’t use my hands all the time and I don’t trigger my tics” [Participant 33].
No bridge to human assistant	Participants recognized that a digital tool would need to allow another person to help them with the app.	“The web page must offer an assistance service so that another person can help in carrying out the tasks defined on the web page” [Participant 97].
Lack of confidentiality	Participants believed that a digital tool would not be confidential enough.	“Confidentiality” [Participant 79].
Cumbersome interface	Participants expressed concerns that a digital tool is difficult to use because it would have too many tasks or options.	“If manipulating the app is difficult...I’m not going to use it” [Participant 77].
Poor educational capacity	Participants expressed concerns that a digital tool would contain inaccurate information or otherwise fail to educate.	“I worry that the app...doesn’t teach me anything” [Participant 69].
Inefficacy	Participants believed that a digital tool may not meet their expectations or be ineffective overall.	“I am concerned that it does not help me improve my tics, that it creates false expectations about whether it can help people with tics” [Participant 87].
Lack of engagement	Participants expressed concerns that a digital tool would be dull or uninteresting.	“It can be slow, boring, and it doesn’t attract attention” [Participant 74].
Poor learnability	Participants expressed concern that they would need to spend a lot of time to understand the digital tool.	“I am concerned that it takes a long time to learn how to use the app” [Participant 87].
Price	Participants believed that a digital tool would be costly.	“I’m somewhat pessimistic about the tech industry...over-charging users for a simple collection of tools” [Participant 10].
Time demands	Participants perceived a digital tool as too time-consuming.	“I think they should develop activities for the app or website that can be done in a short time, so I don’t worry about my tics” [Participant 35].
Trigger tics	Participants believed that a digital tool would trigger their tics.	“I am concerned about its design, that it includes strong colors or that it does not consider other characteristics that could somehow trigger a tic attack” [Participant 104].

## Discussion

### Summary of Findings and Connections to Prior Work

This study aimed to gather information toward (1) how adults with tics use and perceive available resources for tic management and support, (2) better understanding of the concerns adults with tics have about using technology for tic management and support, and (3) what adults with tics would like technology to do for them. Our findings indicate that adults with tics predominantly look for resources from the internet and find solace in family members; have concerns about future digital tools being complicated, time-consuming to use, and inaccessible; and are looking for tic and trigger monitoring in digital tools.

It is concerning that a social media site such as YouTube was a common and even first source of information for participants as opposed to consulting with professionals in the field. While this finding appears to align with prior work noting difficulties in accessing specialty care [12,13], studies have indicated that TS portrayals on YouTube and other social media may “inadvertently reinforce negative stereotypes and spread misinformation” [29]. It is unfortunately possible that participants are reaching out to these kinds of sources first not only out of convenience but also due to negative experiences with professionals [14].

At first glance, family being a more commonly noted source of support than friends appeared to differ slightly from prior work, which has suggested that individuals aged 16 years or older and who are current users of at least 1 online support community for TDs place considerable importance on being supported by their peers versus family and other forms of support [30]. However, it is to be expected that any study exclusively recruiting from online support communities would yield participants who place greater reliance on online peers.

In this study, tic, trigger, and mood, anxiety, or stress monitoring were ranked as the 3 most desired features in a digital tool. In contrast, prior work has reported that young people with TS commonly consider the following as desired features of a digital tool: “ways to learn more about TS” (including interaction with others), “activities that help to keep the [young person] calm” (such as games or music), and “functions that help them organize their activities” (such as a scheduling tool) [31]. The results from this study appear to suggest that adults with tics believe more in the value of monitoring themselves and their individual situation as opposed to learning by interacting with others. Self-monitoring tics and triggers are known by the medical and research communities to be part of effective therapeutic activities for TDs [32,33], so it is a hopeful result that participants saw the value in these features. By gathering systematic data on the frequency and correlates of their tics, individuals might be better able to design personalized strategies for mitigating their influence similar to the function-based interventions central to behavior therapy for tics. Stress, too, has traditionally been associated with increased tic severity [34] and so tools to identify and manage stress could result in overall reductions in tic severity. Relaxation therapy has also been found to be significantly effective in decreasing the severity or the frequency of tic symptoms, although not as much as self-monitoring and habit reversal [35]. Empirical testing would be necessary to determine whether self-directed use of a digital tool would be sufficient to produce clinical gains or whether it would be better conceived as an adjunct to clinical care.

Intriguingly, our study participants for the most part did not engage with tools specifically for tic management, citing that such tools were not available. Given that tic and trigger monitoring were popular choices as the top requested feature, this finding suggests that the currently existing tools were either not well known or did not support users enough to be useful. Instead of tools directly related to tics, participants relied heavily on tools for mental health support. One user also noted difficulty with self-monitoring tics or triggers: “I believe I tried tracking tics long ago but it was just too frequent to keep up with it and there were no consistent triggers to record” [Participant 14].

The findings here suggest that even when our study participants are familiar with certain tic-related tools, they have come away lacking, often due to user experience issues related to engagement or accessibility. However, they have found benefits from other apps that provide mental health and community support. The majority (115/153, 75.2%) are enthusiastic about new tools being developed to more specifically help them manage their tics and day-to-day life, but simultaneously some (42/158, 26.6%) express concern that such tools would not ultimately be able to help them.

### Limitations

Our study has several limitations that should be considered. First, technology changes quickly. It is possible that participants would now have new thoughts, desired features, or concerns relevant to digital tools for TDs with the proliferation of generative artificial intelligence models and other advances.

To limit participant burden and prevent fatigue effects, we did not include any formal assessment of current tic symptomology, such as the Adult Tic Questionnaire [36], which would have substantially increased item count. We acknowledge that this may limit comparability with studies that use that instrument.

We used a 14-item ranking section for item 6 of our questionnaire, which asked participants to rank possible features of a digital tool from most to least important. While this question was designed to better understand the features that adults with tics were most interested in, it is possible that the number of items contributed to survey fatigue. In addition, 1 participant reported that they found the survey difficult to complete. A future version of this instrument might instead have respondents rate each of the 14 items on a Likert scale (eg, from “Very Unimportant” to “Very Important”). Future work might also look into face validation of the survey instrument through pilot testing or personal interviews.

Our participants consisted of volunteers and may also show an increased tendency to rely on online resources and tourette.org, specifically, as we recruited participants in part via this website. Furthermore, it is possible that those who answer an online survey are more likely to endorse a digital means of capturing or receiving information.

We note, too, that our participant pool consisted only of adults with tics, and people younger than 18 years may have differing perspectives. In addition, we do not claim that the results presented here are entirely exhaustive of understanding the TDs community as a whole, and tics and related needs may vary greatly by individual. However, these data represent a key step toward better understanding the current user experience for individuals with tics and the next steps toward supporting them.

### Broader Considerations

We were able to reach our study participants through state and local chapters as well as through an advertisement on the TAA website and expect that a future tool, if effective, could be disseminated through community groups in a similar way. Ideally, such an app would also be designed in collaboration with an organization of repute (such as the TAA) to ensure that any information or support it provides is accurate and trustworthy, in line with the concerns expressed by our study participants. Collaboration with a user experience professional would also be crucial, given that participants overwhelmingly feared that such an app would not adhere to recognized usability standards. In addition, we recommend that experts in data security as well as legal and regulatory compliance be involved, given the concern for data confidentiality. Risk assessments, as well as reviews to maintain compliance with common standards (eg, HITRUST Common Security Framework), should be conducted regularly.

Overall, people with tics, and particularly adults with tics, appear to be underserved when it comes to digital tools. We hope this formative work serves as a first step toward future human-centered technologies for adults with tics.

## Supplementary material

10.2196/78328 Multimedia Appendix 1 Full questionnaire items and response formats used in the study.

10.2196/78328 Checklist CHERRIES checklist.

## Abbreviations

CHERRIES: Checklist for Reporting Results of Internet E-Surveys
TAA: Tourette Association of America
TD: tic disorder
TS: Tourette syndrome
