# Supplementary material for: The Role of Digital Tools in Meeting the Needs of Adults With Tourette Syndrome: A Human-Centered Design Approach
Source: JMIR Form Res. 2026 Feb 19;10:e78328. doi: 10.2196/78328 (PMC12919968; doi:10.2196/78328)
Supplement: Multimedia Appendix 1 [file formative-v10-e78328-s001.pdf]

Multimedia Appendix 1. Survey Instrument

Full questionnaire items and response formats used in the study.

Legend

CE = Consent-Eligibility
D = Demographics
Q = Questionnaire
CR = Consent-Recontact

[Consent-Eligibility]

CE1. Please choose one of the following.

Response format: single-select (radio buttons)

Options:

- I certify that I am 18 years of age or older, I have read and understand the information above, and I freely consent to participate in this study.

- I do not wish to participate in this study.

Order: options listed in order shown above.
Default: no preselection.
Other/Specify: no.
Required: yes.

Branching:
- If “I certify that…” → continue to D1
- If “I do not wish to participate…” → continue to CR1 (exit message).

[Demographic Information]

Introductory text shown to all respondents: Please begin by telling us a bit about yourself.

D1. How old are you?

Response format: text field (single line, 500 chars).

Required: no

D2. What is your gender?

Response format: single-select (radio buttons) with an “Other” option that allows text entry.

Options:
- Man

- Woman
- Non-binary
- Other (please describe): ____

Order: options listed in order shown above.
Default: no preselection.
Other/Specify: yes. Text field: single line (500 chars).
Required: yes (participants may select “Prefer not to say” to proceed).

D3. What is your racial identity?

Response format: single-select (radio buttons) with an “Other” option that allows text entry.

Options:
- American Indian or Alaska Native
- Asian
- Black or African-American
- Native Hawaiian or Other Pacific Islander
- White
- More than one race
- Other (please describe): ____
- Prefer not to say

Order: options listed in order shown above.
Default: no preselection.
Other/Specify: yes. Text field: single line (500 chars).
Required: yes (participants may select “Prefer not to say” to proceed)

D4. Are you of Hispanic origin?

Response format: single-select (radio buttons)

Options:
- Yes
- No
- Not sure
- Prefer not to say

Order: options listed in order shown above.
Default: no preselection.
Other/Specify: no.
Required: yes (participants may select “Prefer not to say” to proceed)

D5. Where do you currently live?

Response format: single-select (drop-down)

Options:
- Alabama
- Alaska
- Arizona

- Arkansas
- California
- Colorado
- Connecticut
- Delaware
- District of Columbia
- Florida
- Georgia
- Hawaii
- Idaho
- Illinois
- Indiana
- Iowa
- Kansas
- Kentucky
- Louisiana
- Maine
- Maryland
- Massachusetts
- Michigan
- Minnesota
- Mississippi
- Missouri
- Montana
- Nebraska
- Nevada
- New Hampshire
- New Jersey
- New Mexico
- New York
- North Carolina
- North Dakota
- Ohio
- Oklahoma
- Oregon
- Pennsylvania
- Rhode Island
- South Carolina
- South Dakota
- Tennessee
- Texas
- Utah
- Vermont
- Virginia
- Washington
- West Virginia
- Wisconsin
- Wyoming

Order: options listed in alphabetical order.
Default: no preselection.

Other/Specify: no.
Required: no

D6. What age did your tics begin?
Response format: text field (single line, 500 chars).
Required: no.

D7. Have you been formally diagnosed by a healthcare provider with any of the following?

Response format: multi-select (checkboxes)

Options:
- Tourette Syndrome
- Chronic or Persistent Tic Disorder
- Transient Tic Disorder
- Other (please describe): ____
- None of the above

Order: options listed in order shown above.
Default: no preselection.
Other/Specify: yes. Text field: single line (500 chars).
Required: no

[Questionnaire - Information about Tics]

Introductory text shown to all respondents: The next few questions are about where you get information about and support for your tics.

Q1. In the past, where have you gotten information about tics? Please select ALL that apply.

Response format: multi-select (checkboxes) with an "Other" option that allows text entry.

Options:
- Primary Care Physician
- Neurologist
- Psychiatrist
- Psychologist
- Social Worker
- Occupational Therapist
- Physical Therapist
- School Counselor
- Books
- YouTube
- Google
- Wikipedia
- Hospital Websites (e.g., the Mayo Clinic, the Cleveland clinic)
- WebMD
- Tik Tok
- Facebook
- Instagram
- Twitter

- The Tourette Association of America website (tourette.org)
- Reddit
- Other (please describe): ____

Order: options listed in random order.
Default: no preselection.
Other/Specify: yes. Text field: single line (500 chars).
Required: yes.

Q2. If you had a question about tics, where is the first place you would go to get an answer? Please choose only ONE answer.

Response format: single-select (radio buttons) with an “Other” option that allows text entry.

Options:
- Primary Care Physician
- Neurologist
- Psychiatrist
- Psychologist
- Social Worker
- Occupational Therapist
- Physical Therapist
- School Counselor
- Books
- YouTube
- Google
- Wikipedia
- Hospital Websites (e.g., the Mayo Clinic, the Cleveland clinic)
- WebMD
- Tik Tok
- Facebook
- Instagram
- Twitter
- The Tourette Association of America website (tourette.org)
- Reddit
- Other (please describe): ____

Order: options listed in random order.
Default: no preselection.
Other/Specify: yes. Text field: single line (500 chars).
Required: yes.

[Questionnaire - Treatment and Support]

Q3. Have you ever received any of the following treatments for your tics?

Response format: multi-select (checkboxes) with an “Other” option that allows text entry.

Options:
- Medication
- Comprehensive Behavioral Intervention for Tics (CBIT) or Habit Reversal Therapy (HRT)

- Exposure and Response Prevention (ERP)
- Other therapy
- Deep Brain Stimulation
- Other (please describe): ____

Order: options listed in order shown above.
Default: no preselection.
Other/Specify: yes. Text field: single line (500 chars).
Required: no.

Q4. Where do you receive support for your tics? Support might include treatment, accommodations at school or work, encouragement, understanding, and more. It's basically anything that helps you manage or feel better about your tics. Please choose all that apply.

Options:

- Coach
- Counselor
- Co-workers
- Educator (e.g., teacher or academic administrator)
- Employer
- Family members
- Fitness instructor
- Friends
- Health care provider
- Local support group
- Meditation teacher
- Online communities or message boards (please name, if comfortable doing so): ____
- Spouse or partner
- Other (please describe): ____

Order: options listed in random order.
Default: no preselection.
Other/Specify: yes, for two options as shown. Text field in each case: single line (500 chars).
Required: no.

Q5. Are there any forms of support that you wish you had, but don't?

Response format: text field (multiple lines, 20000 chars).
Default: none.
Required: no.

[Questionnaire - Digital Tool Questions]

Introductory text shown to all respondents: The next few questions are about what you imagine being helpful in a digital tool for tics.

Q6. A digital tool can do lots of different things. Please consider the list below and rank order the various features from most to least important. You can move the features by clicking on them and dragging them to the appropriate spot.

Response format: rank order (drag and drop)

Options (to be ranked):
- Tic monitoring (e.g., a way to monitor how often your tics occur and see how they change over time)
- Mood/Anxiety/Stress monitoring (e.g., a way to monitor your emotional states and see how they change over time)
- Stress reduction/Relaxation strategies (e.g., progressive muscle relaxation, physical exercise)
- Reminders to practice skills you have learned in therapy (e.g., awareness training,
- competing responses)
- Reminders to take medicine
- Information about tics (e.g., how common they are, what causes them)
- Ways to connect with others who have tics (e.g., a message board)
- An opportunity to share your story with others who have tics
- Tools for managing negative thoughts you may have about your tics
- Mindfulness-based tools (e.g., meditation, breathing exercises, yoga)
- Encouragement
- Tips for talking about tics with others
- Anger management strategies
- Trigger monitoring (e.g., a way to track factors that make your tics better or worse)

Order: options listed in random order.
Default: none.
Ties: not allowed (strict ordinal ranking).
Requirement: full ranking (all options must be placed).
Presentation: numbered drop targets (1 = most important).
Required: no..

Q7. Are there any other things that you would like a digital tool to do for you?

Response format: text field (multiple lines, 20000 chars).
Default: none.
Required: no.

Q8. How do you prefer to navigate on your digital devices? You can rank order the choices below from most to least preferred.

Response format: rank order (drag and drop)

Options (to be ranked):
- Trackpad
- Mouse
- Keyboard
- Screen
- Voice commands

Order: options listed in random order.
Default: none.
Ties: not allowed (strict ordinal ranking).
Requirement: full ranking (all options must be placed).

Presentation: numbered drop targets (1 = most important).
Required: no.

Q9. Do your tics make it difficult or frustrating for you to use a computer, smartphone, tablet, or other digital device?

Response format: single-select (radio buttons)

Options:

- Yes
- No

Order: options listed in order shown above.
Default: no preselection.
Other/Specify: no.
Required: no.

Branching:

- If “Yes” → continue to Q10.
- If “No” → continue to Q11.

Q10. How do your tics make it difficult or frustrating to use digital devices?

Response format: text field (multiple lines, 20000 chars).
Default: none.
Required: no.

Q11. Have you ever used a digital tool to help with your tics or any other aspect of your health?

Response format: single-select (radio buttons)

Options:

- Yes
- No

Order: options listed in order shown above.
Default: no preselection.
Other/Specify: no.
Required: no.

Branching:

- If “Yes” → continue to Q12.
- If “No” → continue to Q13.

Q12. That's great! Could you tell us a little bit about the tool(s) you have used?

We'd love to know the name(s) of any tools you have used and what you liked and didn't like about them.

Response format: text field (multiple lines, 20000 chars).
Default: none.

Required: no.

Q13. Do you think a digital tool designed for adults with tics, specifically, could be helpful?

Response format: single-select (radio buttons)

Options:
- Yes
- Possibly
- Unsure
- Unlikely
- No

Order: options listed in order shown above.
Default: no preselection.
Other/Specify: no.
Required: yes (participants may select “Unsure” to proceed).

Q14. How likely would you be to use a digital tool for adults with tics?

Response format: single-select (linear scale)

Options:
- Very likely
- Somewhat likely
- Neither likely nor unlikely
- Somewhat unlikely
- Very unlikely

Order: options listed in order shown above linearly, from left to right.
Default: no preselection.
Other/Specify: no.
Required: no.

Q15. Which type of tool would be most appealing to you?

Response format: single-select (radio buttons)

Options:
- Website
- App
- Both
- Neither

Order: options listed in order shown above.
Default: no preselection.
Other/Specify: no.
Required: no.

Q16. Do you have any concerns about using a digital tool for managing your tics?

Response format: single-select (radio buttons)

Options:

- Yes
- No

Order: options listed in order shown above.
Default: no preselection.
Other/Specify: no.
Required: no.

Branching:

- If “Yes” → continue to Q17.
- If “No” → continue to Q18.

Q17. What are your concerns?

Response format: text field (multiple lines, 20000 chars).
Default: none.
Required: no.

[Questionnaire - Additional Comments]

Q18. Is there anything else you'd like to share with us?

Response format: text field (multiple lines, 20000 chars).
Default: none.
Required: no.

[Contact Information and Recontact Consent]

CR1. Thank you very much for completing our survey. We really appreciate the time and energy you put into sharing your thoughts.

To receive your $10 gift card, please enter your email address below. Gift cards will be sent out as soon as possible, but please allow up to one week to receive yours.

If you don't receive one within one week, or if you have any questions or concerns about this study, please contact Hannah Reese, Ph.D. at ticresearch@bowdoin.edu or 207-798-4146.

Options:

- Email address: ____

Response format: text field (single line, 500 chars).
Default: none
Required: no

CR2. Would you like us to retain your email address so we can inform you about future research

studies on this or other topics related to tics?

Response format: single-select (radio buttons)

Options:
- Yes
- No

Order: options listed in order shown above.
Default: no preselection.
Other/Specify: no.
Required: no.
