# Supplementary material for: The Role of Digital Tools in Meeting the Needs of Adults With Tourette Syndrome: A Human-Centered Design Approach
Source: JMIR Form Res. 2026 Feb 19;10:e78328. doi: 10.2196/78328 (PMC12919968; doi:10.2196/78328)
Supplement: Checklist [file formative-v10-e78328-s002.pdf]

CHERRIES Checklist

Completed Checklist for Reporting Results of Internet E-Surveys (CHERRIES) for this study.

Table 1. Checklist for Reporting Results of Internet E-Surveys (CHERRIES).

Category	Item	How Addressed in This Study
Design	Survey design	Cross-sectional descriptive study assessing the attitudes of adults with motor and/or vocal tics who were living in the United States toward current and future digital tools for managing one's tics.
IRB	IRB approval	Reviewed and deemed exempt by the Institutional Review Board [details redacted for blind review] per Exemption 45 CFR 46.104(d)(2) for Educational Tests, Surveys, Interviews, or Observation of Public Behavior. Limited review was conducted to ensure that there were adequate provisions to protect the privacy of subjects and to maintain the confidentiality of data.
	Informed consent	Participants viewed an online information sheet and chose "I certify that I am 18 years of age or older, I have read and understand the information above, and I freely consent to participate in this study" to provide informed consent before accessing the questionnaire.
	Data protection	All data stored on secure, password-protected server; responses anonymized prior to analysis.
Development and pre-testing	Development and testing	Draft survey was piloted on desktop and mobile devices by the authors; minor wording changes made to improve clarity.
Recruitment process	Open survey versus closed survey	Open survey
	Contact mode	Initial contact with the potential participants was made on the Internet
	Advertising the survey	Survey was distributed to adults with motor and/or vocal tics who were living in the United States via an advertisement on the Tourette Association of America (TAA) website (tourette.org). Invitation emails from the authors were also sent to state and local TAA chapters and support groups as well as tic disorder clinicians and researchers.
Survey administration	Web/E-mail	Email survey
	Context	The tourette.org website features resources to raise awareness and provide ongoing support to patients and families impacted by Tourette Syndrome and tics.

	Mandatory / voluntary	Voluntary
	Incentives	Participants received a $10 Amazon gift card for their participation.
	Time / Date	Data collected between December 2022 and April 2023
	Randomization of items or questionnaires	Answer choice randomization was applied to questionnaire items Q1, Q2, Q4, Q6, and Q8.
	Adaptive questioning	3 conditionally displayed questionnaire items based on responses to reduce number and complexity of the questions
	Number of Items	7 demographic information items and up to 18 questionnaire items
	Number of screens (pages)	9
	Completeness check	This survey was created with Qualtrics which offered completeness check features, including automatically storing survey progress as respondents move between pages.
	Review step	Respondents were able to review and change their answers by navigating back through the survey.
Response rates	Unique site visitor	Unique visitors determined via IP address
	View rate	169
	Participation rate	Unknown (due to recruitment through organizations and groups)
	Completion rate	152/168 fully completed; 158/168 fully or partially completed
Preventing multiple entries from the same individual	Cookies used	Yes; Qualtrics was used to prevent multiple entries using a cookie-based system.
	IP check	No additional exclusions were performed based on IP address
	Log file analysis	No additional exclusions were performed based on the log file
	Registration	Users were not required to register to complete the survey
Analysis	Handling of incomplete questionnaires	Complete and incomplete questionnaires were included in analysis.

	Questionnaires submitted with an atypical timestamp	Each record was assigned an entry ID ('E' followed by a number) according to the time the survey was completed. We then assigned participant IDs ('P' followed by a number) to those entries that met the criteria to be considered participants.<br><br>Questionnaires submitted in less than 2 minutes were examined (n=10). Of these: one did not wish to participate (E75), one self-reported duplicate was excluded (E18), six that only completed consent form were excluded (E23, E48, E57, E69, E93, E118), and two partial completers were retained (E31, E90).
	Statistical correction	Multiple pairwise correlations (14 features) were tested. A Bonferroni correction was applied to control for the increased possibility of Type I errors ($\alpha = .05/14 = .0036$). Unadjusted p-values are also reported for transparency.

This checklist has been modified from Eysenbach G. Improving the Quality of Web Surveys: the Checklist for Reporting Results of Internet E-Surveys (CHERRIES). J Med Internet Res. 2004 Sep 29;6(3):e34 [erratum in J Med Internet Res. 2012; 14(1): e8.]. Article available at https://www.jmir.org/2004/3/e34/; erratum available https://www.jmir.org/2012/1/e8/. Copyright ©Gunther Eysenbach. Originally published in the Journal of Medical Internet Research, 29.9.2004 and 04.01.2012.
